# Influence of Tempering Temperature and Time on Microstructure and Mechanical Properties of Additively Manufactured H13 Tool Steel

**DOI:** 10.3390/ma15238329

**Published:** 2022-11-23

**Authors:** Kichang Bae, Hyoung-Seok Moon, Yongho Park, Ilguk Jo, Junghoon Lee

**Affiliations:** 1 Department of Metallurgical Engineering, Pukyong National University, Busan 48513, Republic of Korea; 2 Advanced Energy Materials and Components R&D Group, Korea Institute of Industrial Technology, Busan 46938, Republic of Korea; 3 Department of Materials Science and Engineering, Pusan National University, Busan 46241, Republic of Korea; 4 Department of Advanced Materials Engineering, Dong-Eui University, Busan 47340, Republic of Korea

**Keywords:** additive manufacturing, laser powder bed fusion, AISI H13 tool steel, post-heat treatment, tempering

## Abstract

Among various processes for manufacturing complex-shaped metal parts, additive manufacturing is highlighted as a process capable of reducing the wastage of materials without requiring a post-process, such as machining and finishing. In particular, it is a suitable new manufacturing technology for producing AISI H13 tool steel for hot-worked molds with complex cooling channels. In this study, we manufactured AISI H13 tool steel using the laser power bed fusion (LPBF) process and investigated the effects of tempering temperature and holding time on its microstructure and mechanical properties. The mechanical properties of the sub-grain cell microstructure of the AISI H13 tool steel manufactured using the LPBF process were superior to that of the H13 tool steel manufactured using the conventional method. These sub-grain cells decomposed and disappeared during the austenitizing process; however, the mechanical properties could be restored at a tempering temperature of 500 °C or higher owing to the secondary hardening and distribution of carbides. Furthermore, the mechanical properties deteriorated because of the decomposition of the martensite phase and the accumulation and coarsening of carbides when over-tempering occurred at 500 °C for 5 h and 550 °C for 3 h.

## 1. Introduction

Additive manufacturing (AM) is a process of directly fabricating a final product by stacking objects in a layer-by-layer manner using a computer-aided three-dimensional design [1,2,3,4]. With the advent of the fourth industrial revolution, the process is gaining attention as an innovative technology that can change the manufacturing industry because of the easy manufacturing of complex-shaped products that are difficult to manufacture using conventional processes. Metal AM is a process of printing products using metal powder or metal wire. The metal parts produced by AM can be used in various industries, such as the automobile, structural material, aerospace, medical, and remanufacturing fields [5,6,7,8]. Directed energy deposition (DED) and powder bed fusion (PBF) are the commonly used metal AM processes. In particular, the PBF process is primarily used to manufacture multiple small and complex products simultaneously. Among the various energy sources, the laser powder bed fusion (LPBF) process uses a laser beam as a heat source for melting metal powder and demonstrates excellent shape accuracy. Several studies have been conducted on the LPBF process to manufacture excellent final products by combining various process parameters such as laser power, laser scanning speed, and type of metal powder [9,10,11].

AISI H13 tool steel (H13), known as hot-worked mold steel, is widely used in molds with cooling channels, such as die casting, plastic injection molds, forging, and extrusion because of its high-temperature strength, ductility, excellent tempering resistance, and wear resistance [12,13,14]. The H13 mold is conventionally manufactured by the subtractive processing of large H13 blocks. However, due to the high hardness of the H13, it is difficult to create complex shapes and cooling channels by cutting and machining with tools [15,16]. Moreover, the subtractive process incurs a significant cost, time, and wastage of material. In contrast, the LPBF process can overcome these limitations. Complex-shaped molds and cooling channels in molds, which improve the quality of the product and the lifetime of the mold, can be created using the LPBF process of mold metals. These advantages of the LPBF process for applications to metal mold fabrication have triggered extensive studies on the optimization of LPBF of mold metals.

Problems such as porosity, cracking, and high residual stresses can occur due to rapid cooling (approximately 10^6–7^ K/s) during the manufacturing of H13 using the AM process [17,18,19]. To solve these problems, various post-treatment processes using hot isostatic pressing (HIP) and heat treatment (HT) have been developed. HT, especially, can efficiently resolve these problems via microstructure changes. Therefore, the effects of the process parameters of AM and post-HT on the microstructure and mechanical properties of H13 manufactured using the AM process need to be understood. Several studies have been conducted to analyze the microstructure and mechanical properties of H13 manufactured using AM and the effects of post-HT. Åsberg et al. [20] studied the effect of post-treatment on the microstructure, pores, and mechanical properties of H13 manufactured using the LPBF process. They suggested that post-treatment using HIP could reduce pores and fusion defects, thereby improving the mechanical properties. Yuan et al. [21] reported the effect of the post-treatment on the tensile and impact toughness properties of H13 manufactured using the LPBF process. They revealed that the direct tempering of H13, showing more dispersal of carbide, resulted in a lower thermal softening than conventional post-HT of H13, which includes a solution treatment before the tempering. Wang et al. [22] investigated the effect of a double-tempering HT on the thermal fatigue properties of H13 manufactured using a selective laser melting (SLM) process, which fully melts the metal powder. The H13 manufactured using the SLM showed a superior thermal fatigue resistance compared to the forged H13 due to its fine microstructure and higher amount of retained austenite. The relationship between the microstructure changes by post-HT and the mechanical properties of the H13 manufactured using the AM process has been extensively explored. Evaluation of various post-HT processes is crucial in the practical applications of the H13 manufactured by the AM process.

In this study, we applied various tempering temperatures and times to H13 steel manufactured using SLM, which is one of the LPBF processes, then the effects of tempering temperature and time on microstructure and mechanical properties were investigated by comparing as-built. Tempering temperature and time were used as variables after the solution treated H13.

## 2. Materials and Methods

Based on the PBF process, specimens were printed using the LPBF process using Mlab Cushing R (Concept Laser, GE, Boston, MA, USA) with Nd:YAG fiber laser (wavelength: 1064 nm) as a heat source. Spherical gas-atomized H13 steel powder (Sandvik Osprey Ltd., Sandviken, Sweden) with an average particle diameter of −47 µm was used to fabricate the specimens used in this study. The chemical compositions of H13 steel powder are listed in Table 1.

**Figure 1:**
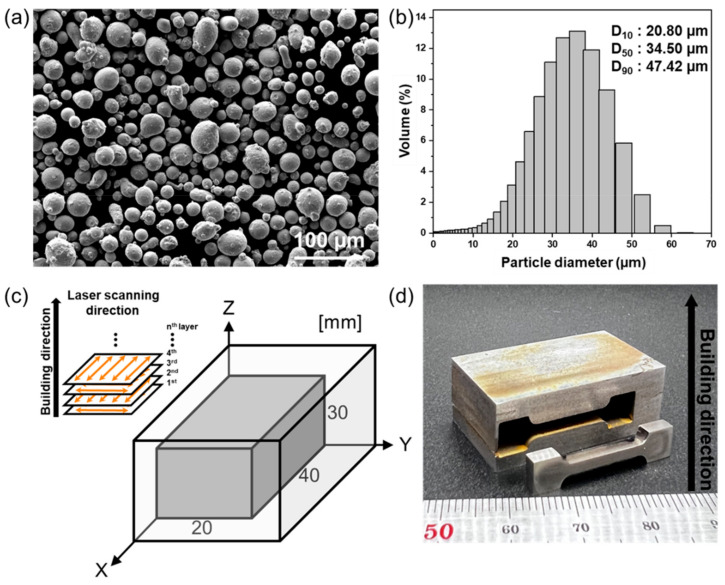
(**a**) Morphology and (**b**) particle size analysis of AISI H13 powder steel. (**c**) Schematic illustrations of the LPBF process and (**d**) printed sample and tensile specimen images.

The morphology of the H13 powder (see Figure 1a) was observed using field-emission scanning electron microscopy (FE-SEM, MIRA 3, Tescan, Brno, Czech Republic), a particle size analyzer (PSA, LS 320, BoulA, BoulAcer) was used to measure the average particle diameter of H13 powder (Figure 1b). An LPBF process was performed in an N_2_ (g) atmosphere with an oxygen concentration of less than 1% to prevent oxidation of the specimen. A 90° rotate scanning strategy was used between each layer (see Figure 1c). The detailed parameters of LPBF used in this study are listed in Table 2.

Specimens were built in a rectangular shape of 20 × 40 × 30 mm^3^ to evaluate the microstructure and mechanical properties of the post-heat treatment process of the H13 tool steel manufactured using the LPBF. Heat treatment was performed in an Ar (g) atmosphere using a horizontal tubular furnace to observe changes in the microstructure and mechanical properties of the post-heat treatment specimen. Solution treatment was performed at 1000 °C for 1 h, and tempering heat treatment was conducted at 200, 300, and 500 °C for 2, 3, and 5 h, respectively. H13 steel is known to cause secondary hardening around 500 °C. Therefore, 200 °C, which is a relatively lower temperature, and 500 °C and 550 °C, which are the temperatures at which secondary hardening occurs, were selected. After solution treatment, the specimen was oil quenched (O.Q.), and the tempering heat-treated specimen was cooled by air cooling (A.C.). Specimen notations according to each heat treatment condition are shown in Table 3.

Phase analysis was analyzed using X-ray diffraction (XRD, Ultima IV, Rigaku, Tokyo, Japan) in the range of 2θ between 10° and 90° using the Mo target (Kα = 0.7093 Å) under the condition of an acceleration voltage of 30 kV, current of 40 mA, and scanning speed of 1.2 °/min. For microstructure observation, micro-polishing was performed down to 1 μm in each specimen, and etching was conducted using Viella’s agent (1 g picric acid, 5 mL HCl, and 95 mL ethanol). The microstructure of each specimen was observed using FE-SEM.

Hardness and tensile tests were performed to study the mechanical properties of the H13 manufactured using the LPBF process. For the hardness test, a Digital Rockwell hardness tester (MV-1, MATSUZAWA, Akita, Japan) was used, 10 hardness measurements were carried out for each specimen, and an average value was used. A universal testing machine (RB301, UNITECH-T, R&B Inc., Daejeon, Republic of Korea) was used to evaluate tensile properties. Tensile specimens were machined in a direction perpendicular to the built direction from specimens prepared using the H13 manufactured by the LPBF process (Figure 1d) with ASTM E8 standard for plate-type sub-sized specimens (gauge length = 13.2 mm, thickness = 2 mm). Micro-polishing of the tensile specimen was performed down to 1 μm. A tensile test was conducted at a strain rate of 1.0 × 10^−3^ s^−1^, and strain gauges were attached to the specimens for elongation measurements. A tensile test was conducted in the atmospheric atmosphere at room temperature, and FE-SEM was used for fracture surface observation after the tensile test.

To minimize the experimental error in hardness, elongation, and tensile strength, we used seven samples fabricated by each condition. Excluding maximum and minimum values, five results were averaged. Then, stress–strain curves, which have the most similar values (i.e., ultimate tensile strength and elongation) to average, were shown as representative results.

## 3. Results and Discussion

**Figure 2:**
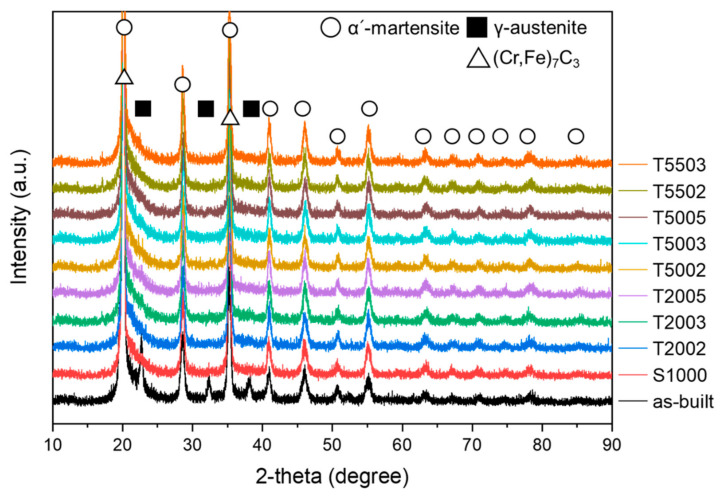
XRD analysis of each specimen according to the heat treatment conditions.

XRD analysis was performed to examine the phase changes according to the HT conditions, and the results are shown in Figure 2. The peaks of the α’-martensite and (Cr,Fe)_7_C_3_ carbide (M_7_C_3_) are observed in all specimens. The M_7_C_3_ is speculated to form in the H13 matrix because of the rapid solidification during the LPBF process. M_7_C_3_ has a hexagonal close-packed structure. These carbides are thermally unstable at high temperatures and easily precipitate, decompose, and grow [23]. Conversely, the γ-austenite phase is observed only in the as-built specimen, which corresponds to the retained austenite in the matrix due to the rapid cooling during the LPBF process. These retained austenite indicate the incomplete transformation from austenite to martensite during the cooling of as-built H13 [24,25]. This peak related to the γ-austenite phase is not observed in the post-HT samples, indicating that the γ-austenite phase disappears due to the solution treatment.

**Figure 3:**
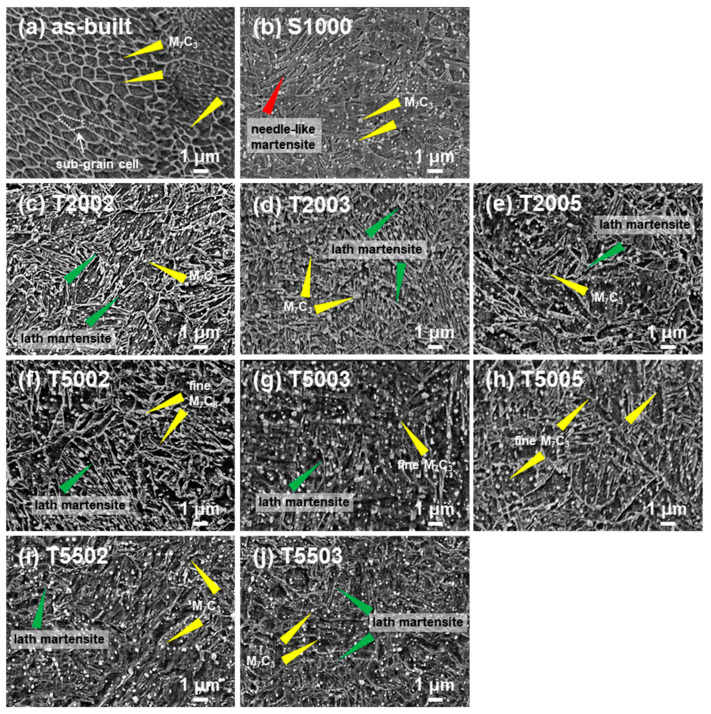
FE-SEM images of the microstructure of each specimen according to the heat treatment conditions.

The changes in the microstructure owing to the post-HT were analyzed using the FE-SEM (Figure 3). The as-built specimen shows the sub-grain cells with a size of less than 2 µm (Figure 3a). These are the specific microstructures that appear during the AM process and have been reported to improve mechanical strength [17,26,27]. These sub-grain cells are α´-martensite [28]. A spherical phase is observed inside the H13 matrix (yellow triangle in Figure 3a), which corresponds to the M_7_C_3_ based on the XRD analysis. In contrast, the sub-grain cells are not observed in the S1000 specimen (Figure 3b), indicating that they decompose and disappear by diffusion during solution treatment. Moreover, a needle-like martensite is observed in the microstructure, indicating that γ-austenite was fully phase-transformed into needle-like α´-martensite during the cooling after the solution treatment. These results are in agreement with the XRD analysis (Figure 2). In addition, white spherical M_7_C_3_ are observed in the as-built specimen, and the size and distribution of spherical M_7_C_3_ are increased during the cooling after the solution treatment. Figure 3c–j show the microstructure of the tempering-treated specimens at various temperatures and holding times after solution treatment. Regardless of tempering temperature and time, the needle-like α´-martensite is changed to a lath martensite phase. However, according to the temperature and time of tempering, significant differences in the M_7_C_3_ precipitates can be observed. Compared with S1000 (as-built H13 with solution treatment and oil-quenching), the specimen with the tempering at 200 °C for 2, 3, and 5 h show a similar distribution of M_7_C_3_ precipitates, but a bigger size of M_7_C_3_ precipitates, indicating a growth of carbides without any new initiation of precipitates during the tempering. In contrast, the specimens with the tempering at more than 500 °C (i.e., T5002, T5003, T5005, T5502, and T5503) have bigger and more M_7_C_3_ precipitates compared to S1000. During the tempering, the M_7_C_3_ precipitates are grown by the diffusion of carbon from the lath martensite, so H13 with the tempering has bigger M_7_C_3_ precipitates. However, in the case of the tempering over 500 °C, the diffusion of carbon is significantly enhanced; thus, M_7_C_3_ precipitates can be nucleated inside of lath martensite grains, accompanying their growth. Therefore, T5503 shows the biggest and most M_7_C_3_ precipitates.

**Figure 4:**
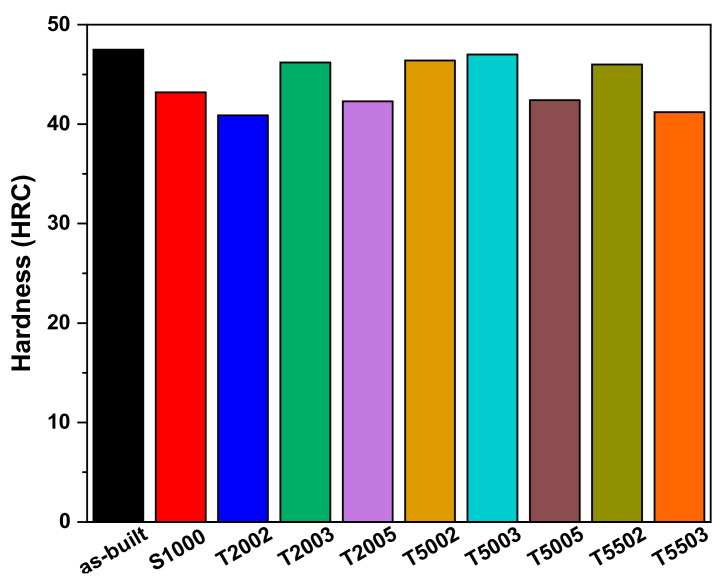
Average hardness of each specimen according to the heat treatment conditions.

The hardness and tensile tests were conducted to investigate the effects of these microstructure changes on the mechanical properties of H13 manufactured using the LPBF process. Figure 4 shows the hardness test results of the H13 manufactured using the LPBF process at different tempering temperatures and times. Among the H13 manufactured using the LPBF process, the as-built specimen shows the highest hardness of 47.5 HRC. The high hardness is attributed to the sub-grain cells and the M_7_C_3_ formed during the AM process [29]. The hardness of S1000 decreases to 43.2 HRC due to the decomposition and disappearance of the sub-grain cells during the austenitizing HT. The specimens T2002, T2003, and T2005, tempered at a low temperature of 200 °C, show a reduction in hardness, suggesting that secondary hardening does not occur at low temperature. A slight increase in the hardness is observed with the increasing holding time at the tempering temperature of 200 °C. The hardness of T5002, T5003, T5005, T5502, and T5503 specimens, where secondary hardening occurs, are observed to be different at different tempering treatment times. The T5002, T5003, and T5502 specimens recover their hardness to 46, 46.4, and 47 HRC, respectively, which is similar to that of the as-built specimen because of the secondary hardening during the tempering process; however, the hardness of T5005 and T5503 decrease to 42.4 and 41.2 HRC, respectively. Therefore, the H13 manufactured using the LPBF process is considered over-tempered at tempering conditions of 500 °C for 5 h and 550 °C for 3 h. This is because of the gathering and coarsening of the finely dispersed M_7_C_3_ under these tempering HT conditions [30,31,32].

**Figure 5:**
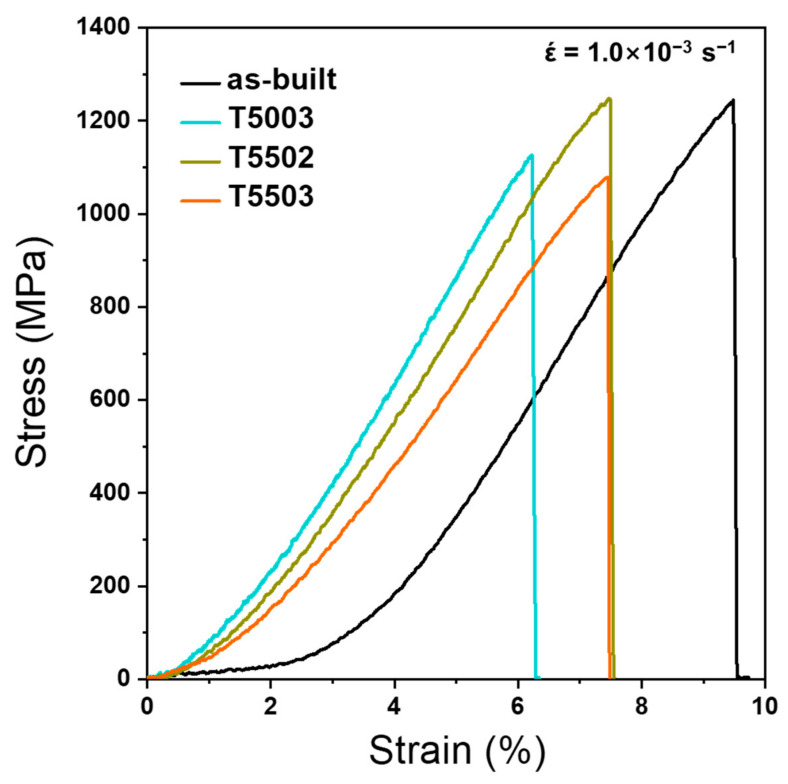
Tensile stress–strain curves of the as-built, T5003, T5502, and T5503 specimens.

**Figure 6:**
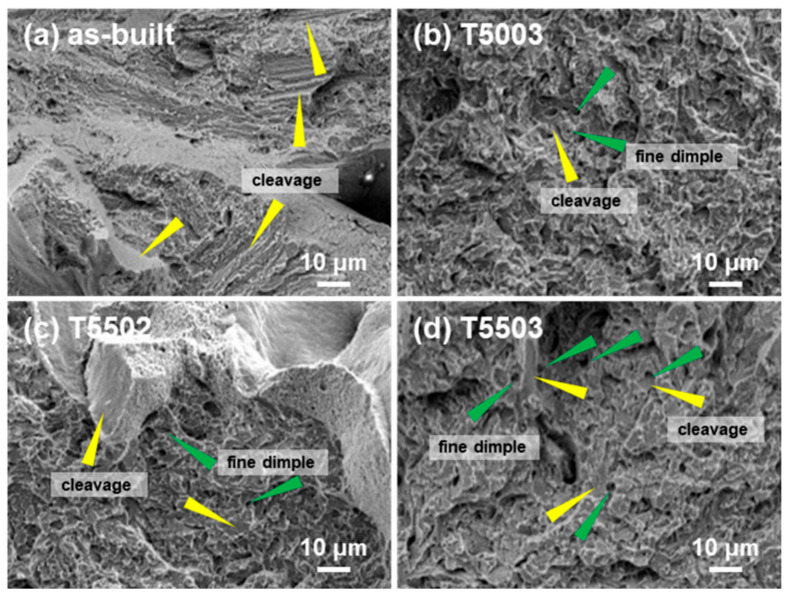
Fracture surface of (**a**) as-built, (**b**) T5003, (**c**) T5502, and (**d**) T5503 specimens.

Tensile tests of the T5003, T5502, and T5503 specimens, selected based on the hardness test results, were conducted, and results were compared with those of the as-built specimens. Figure 5 and Figure 6 show the stress–strain curve and the fracture surface of the as-built, T5003, T5502, and T5503 specimens, respectively. Regardless of the post-HT, all tensile specimens undergo fractures before any plastic deformation occurs. The as-built specimen shows an ultimate tensile strength (UTS) of 1245 MPa and a strain of 9.49% (Figure 5a), thereby suggesting the best tensile properties. Only a cleavage fracture (yellow triangle in Figure 6a) is observed, indicating a typical brittle fracture [33,34]. The hardness of T5003 is similar to that of the as-built specimen; however, the UTS is 1126 MPa, which is 90% of that of the as-built specimen. Furthermore, fine dimples (green triangle in Figure 6b) are observed around the cleavage fracture in the fracture surface of the T5003 (Figure 6b) [35], which is different from that of the as-built specimen. These fine dimples are produced as fracture progress around the M_7_C_3_. When tempering is performed at 500 °C, the secondary hardening does not sufficiently progress, thereby resulting in a fracture at a low UTS during the tensile test. Conversely, the UTS of the T5502 specimen, which has a similar hardness to the as-built specimen, is 1246 MPa; however, the strain decreases to 7.49%. In addition, the fracture surface of the T5502 is observed to primarily contain the cleavage fracture (Figure 6c), similar to the as-built specimen. However, a few fine dimple fractures are also observed, attributed to the improved UTS of the T5502 because of the sufficient secondary hardening during tempering treatment at 550 °C for 2 h. The strain of the T5503 specimen is 7.46%, similar to that of the T5502, whereas the UTS decreases to 1078 MPa. The fracture surface demonstrates a fractography similar to that of the T5003. The observations indicate the decomposition of the lath martensite and coarsening of the finely dispersed M_7_C_3_ during the tempering at 550 °C for 3 h. The H13 manufactured using the LPBF process maintained the highest hardness and tensile strength, which are similar to those of the as-built specimen when post-HT condition of T5502.

Commercial forged H13 steels have been reported to have UTS of 1200–1590 MPa, elongation of 6–9%, and hardness of 46–50 HRC [22,36,37,38]. Regarding these mechanical properties of commercially forged H13, additively manufactured H13 using the LPBF process did not show significant differences in this study. Nevertheless, it should be noted that the LPBF process has an advantage in complex shape fabrication with metals. Therefore, even though the LPBF process cannot significantly improve the mechanical properties of H13, understanding the effects of post-HT in microstructure and mechanical properties of additively manufactured H13 would be valuable in designing and producing metal molds with H13 using the LPBF process.

## 4. Conclusions

H13 manufactured using the LPBF process showed superior mechanical properties without post-HT due to the sub-grain cells. The sub-grain cell structure decomposed and disappeared during solution treatment. A significantly lower hardness was observed when the H13 was tempered at 200 °C than the as-built specimen, which can be attributed to the insufficient secondary hardening at low temperature. When tempering was performed at 500 °C and 550 °C, the hardness could be recovered, indicating that a sufficiently high temperature is required for secondary hardening. In addition, the M_7_C_3_ formed during the LPBF process was finely dispersed in the matrix at a tempering temperature of 500 °C or higher, thereby improving the mechanical properties of the H13. However, when tempering was performed at 500 °C for 5 h or at 550 °C for 3 h, the M_7_C_3_ gathered and coarsened, resulting in a deterioration of the mechanical properties. Therefore, when a post-HT process is designed to improve the mechanical properties of H13 manufactured using the LPBF process, the microstructure changes and secondary hardening by the M_7_C_3_ should be fully understood.

## Figures and Tables

**Table 1:** Chemical compositions of H13 tool steel.

Elements	Fe	Cr	Mo	Mn	Si	V	C
wt.%	Bal.	5.13	1.60	0.47	1.08	1.15	0.41

**Table 2:** Parameters used for the LPBF process of H13 tool steel.

Parameters	Values
Laser power (W)	90
Scanning speed (mm/s)	800
Lamination thickness (mm)	0.025
Overlap (%)	30

**Table 3:** Various post-heat treatment conditions of the H13 manufactured by the LPBF process.

Sample Name	Solution Treatment Temperature (°C)	Time (h)	Cooling	Tempering Temperature (°C)	Time (h)	Cooling
as-built	-	-	-	-	-	-
S1000	1000	1	O.Q.	-	-	-
T2002	1000	1	O.Q.	200	2	A.C.
T2003	1000	1	O.Q.	200	3	A.C.
T2005	1000	1	O.Q.	200	5	A.C.
T5002	1000	1	O.Q.	500	2	A.C.
T5003	1000	1	O.Q.	500	3	A.C.
T5005	1000	1	O.Q.	500	5	A.C.
T5502	1000	1	O.Q.	550	2	A.C.
T5503	1000	1	O.Q.	550	3	A.C.
